# The use of a novel deer antler decellularized cartilage-derived matrix scaffold for repair of osteochondral defects

**DOI:** 10.1186/s13036-021-00274-5

**Published:** 2021-09-03

**Authors:** Wenhui Chu, Gaowei Hu, Lin Peng, Wei Zhang, Zhe Ma

**Affiliations:** 1 School of Life Science, Taizhou University, 1139 Shifu Avenue, Jiaojiang District, Zhejiang 318000 Taizhou, China; 2 Post-Doctoral Innovation Site, Jinan University Affiliation, Yuanzhi Health Technology Co, Ltd, Hengqin New District, 519000 Zhuhai, Guangdong China; 3 Medical Imaging Center, The First Affiliated Hospital of Jinan University, Jinan University, 613 Huangpu Avenue West, Tianhe District, Guangdong 510080 Guangzhou, China

**Keywords:** Decellularization, Antler cartilage, Cartilage-derived matrix scaffolds, Tissue engineering, Osteochondral defect

## Abstract

**Background:**

The physiologic regenerative capacity of cartilage is severely limited. Current studies on the repair of osteochondral defects (OCDs) have mainly focused on the regeneration of cartilage tissues. The antler cartilage is a unique regenerative cartilage that has the potential for cartilage repair.

**Methods:**

Antler decellularized cartilage-derived matrix scaffolds (adCDMs) were prepared by combining freezing-thawing and enzymatic degradation. Their DNA, glycosaminoglycans (GAGs), and collagen content were then detected. Biosafety and biocompatibility were evaluated by pyrogen detection, hemolysis analysis, cytotoxicity evaluation, and subcutaneous implantation experiments. adCDMs were implanted into rabbit articular cartilage defects for 2 months to evaluate their therapeutic effects.

**Results:**

AdCDMs were observed to be rich in collagen and GAGs and devoid of cells. AdCDMs were also determined to have good biosafety and biocompatibility. Both four- and eight-week treatments of OCDs showed a flat and smooth surface of the healing cartilage at the adCDMs filled site. The international cartilage repair society scores (ICRS) of adCDMs were significantly higher than those of controls (porcine dCDMs and normal saline) (*p* < 0.05). The repaired tissue in the adCDM group was fibrotic with high collagen, specifically, type II collagen.

**Conclusions:**

We concluded that adCDMs could achieve excellent cartilage regeneration repair in a rabbit knee OCDs model. Our study stresses the importance and benefits of adCDMs in bone formation and overall anatomical reconstitution, and it provides a novel source for developing cartilage-regenerating repair materials.

**Supplementary Information:**

The online version contains supplementary material available at 10.1186/s13036-021-00274-5.

## Introduction

Healthy bones have the ability to auto-regenerate. However, large defects in the bone structure due to trauma, congenital deformities, or extensive oncological surgery often require surgical reconstruction [1]. Osteochondral defects (OCDs) are focal areas of articular cartilage damage, resulting in the loss of cartilage and inflammation of the adjacent subchondral bone [2]. The most commonly affected joint is the knee, with the majority of lesions located in the femoral condyle and/or patellofemoral articulation [3]. OCDs can lead to discomfort and can have significant impacts on the function of patients [4, 5]. OCDs can cause significant pain, discomfort and reduce the ability to perform activities of daily living. Currently, joint replacement surgery is the only salvage therapy for patients with knee joint systemic osteoarthritis. Since cartilages, except antler cartilage [6], are hypocellular, aneural, and avascular tissues, their regenerative capacity is severely limited. OCDs lead to a poor intrinsic capacity to regenerate healthy cartilage tissue, and many studies are trying to address this.

Cartilage-derived matrix scaffolds (CDMs) have shown great chondrogenic potential in *in vitro* experiments. The results showed that abundant new glycosaminoglycan (GAG)- and collagen type II-containing cartilaginous matrix were formed in CDMs by cultured mesenchymal stromal cells [7]. The potency was further underscored by *in vivo* studies in small animal models both at ectopic [8, 9] and orthotopic locations [10, 11]. The extracellular matrix (ECM) is a natural biomaterial. Decellularization processes can prevent potential immune responses by removing cellular or genetic components. Therefore, issues related to the biocompatibility and biodegradability of ECM are addressed [12]. The advantage of using decellularized CDMs (dCDMs) is that they are of natural origin and can produce bioactive cues. dCDMs can attract and induce cells to differentiate into the appropriate lineages required for tissue repair [13, 14]. Since dCDMs can guide the formation of bone and cartilage tissue, in addition to cartilage repair, these scaffolds have the potential to repair OCDs.

Deer antlers constitute a model organ for examining the regeneration processes of tissues because they are the only mammalian appendages capable of natural regeneration [15]. The basis of antler renewal is dependent on the proliferation and differentiation of antler stem cells (AnSCs), which can maintain the full regeneration of the antler, which occurs yearly, and the cells derived from the progeny can drive an astonishing growth of the antler [16]. The antler, an osseous cranial appendage of male deer, is located on the frontal bone [15]. An antler can generate up to 30 kg of bone tissue at rapid growth rates (up to 2.0 cm/day) [16]. In addition, the cartilage of the antler is vascularized [6]; therefore, endochondral osteogenesis occurs at an incredible speed [17]. Thus, antler cartilage, especially antler decellularized CDMs (adCDMs), could serve as a novel resource for bone repair and cartilage regeneration.

In this study, we aimed to prepare adCDMs that have good biosafety and biocompatibility. In addition, we wanted to demonstrate that adCDMs could achieve excellent cartilage regeneration repair in a rabbit knee OCD model.

## Materials and methods

Animal experiments were carried out at the Animal Experiments Center (Taizhou, China) of the Taizhou University of Medical Sciences under the rules and regulations of the Animal Care and Use Committee of Taizhou University School of Medicine (Permit number: 2019 − 209).

### Pre-treatment of deer antler cartilage and porcine joint cartilage

45-growth-days fresh deer antlers of four-year-old male deer (Shuangyang breed, Chinese sika deer) were collected from Shuangyang (Changchun, China) slaughtering plants (n = 3). The dissection technique described in a previous study was used [18] to prepare the antler cartilage. Under the help of Dr. Chunyi Li, the single-component of cartilage was successfully collected. In brief, the distal 5 cm of the antler tip, which contained AnSCs, was removed and sectioned along the longitudinal axis. The tip was then cut into 5-mm thick slices along the same plane. The slices were further cut into strips measuring 1–2 cm. Under the dissecting microscope, the skin covering the antler slice was removed and washed with phosphate-buffered saline (PBS). Then, all strips were cut into small pieces using two scalpels in a germ-free dish, which were further shattered in a homogenizer (Fluko FA25, liquid-solid ratio = 4:1). Fresh porcine knee joints were obtained from a local market (Taizhou, China). Porcine joint cartilage, cut from the joint surface, was pre-treated using the same protocol as used for the antler cartilages.

### Preparation of antler and porcine decellularized CDMs (adCDMs and pdCDMs)

Deer antler cartilage and porcine joint cartilage were decellularized according to the optimized protocol of Kheir et al. [19] and Utomo et al. [20]. The cartilages were kept dry and subjected to freeze-thaw cycles for six cycles, followed by freeze-thaw cycles at − 80 °C for two days in hypotonic buffer (10 mM Tris-HCl, pH 8.0) after a 24 h incubation in hypotonic buffer at 45 °C. Samples were then treated for 24 h with an ionic detergent, which consisted of 0.1 % SDS, 0.1 % EDTA, and 10 KUI/mL aprotinin in water. Samples were incubated in wash solution (10 KIU/mL aprotinin in PBS) at 45 °C for 24 h after washing twice (30 min each) with wash solution. The samples were then treated with a low concentration elastase solution (0.2 M Tris-HCl, 10 KIU/mL aprotinin, and 0.03 U/mL elastase, pH 8.6) for 24 h at 37 °C. Subsequently, samples were washed twice and incubated at 37 °C in nuclease solution (50 mM Tris-HCl, 10 mM MgCl_2_, 50 µg/mL bovine serum albumin (BSA), 50 U/mL DNase, and 2.5 U/mL RNAse, pH 7.5) for 3 h. Samples were treated in decontamination solution (0.1 % peracetic acid in PBS) for 3 h after washing in wash solution. All incubation and washing steps were performed under continuous stirring. Finally, the samples (pre-washed twice in sterile PBS) were transferred to sterile tubes and incubated in sterile PBS at 45 °C for 24 h. Then, the samples were lyophilized and stored at -20 °C. Samples for histological assays were stored in 4 % formaldehyde. Untreated (i.e., native) cartilage samples were used as controls.

### Preparation of dCDMs derived gel (CDMs-gel)

For gel preparation, we followed the protocol described by Patil et al. [21]. Lyophilized CDMs were crushed into powder and digested in a solution of 0.5 M acetic acid with 10 mg of pepsin (P8160, Solarbio) per 100 mg CDMs for 48 h. Before use, the pH was adjusted to 7.0 using cold 10 M NaOH solution. The pH-adjusted gels, both adCDM-gel and pdCDM-gel, were stored at 4 °C.

### Detection of DNA, GAG, and collagen in adCDMs and pdCDMs

DNA, GAG, and collagen of both adCDMs and pdCDMs were analyzed in this study. adCDMs and pdCDMs were digested at 60 °C overnight in 400 µL and 500 µL papain solution (0.2 M Na_2_H_2_PO_4,_ 0.01 M EDTA·2H_2_O, 250 µg/mL papain, 5 mM L-cysteine, pH 6.0), respectively [22]. DNA content of the adCDMs, pdCDMs, and native cartilages was measured using the Hoechst 33,258 assay (C1017, Beyotime) according to the manufacturer’s instructions. This method can detect low amounts of DNA with a limit of detection at the ng level [23]. A 1,9-dimethylmethylene blue (pH 3.0) assay (HPBIO-JM9048, Hepeng Biology) [24] was used to measure the sulfated GAG content of the CDM scaffolds, and a hydroxyproline assay (A030-3-1, Njjcbio) was conducted to measure the total collagen content of the dCDMs [25].

### Cell viability assessment

The adCDM-derived conditional medium (adCDMs-CM), filtered from adCDMs in dulbecco’s modified eagle medium (DMEM), was used for cell viability assessment, and the CCK-8 (WST-8) assay was performed to evaluate the cytotoxicity of the scaffolds [26]. Rabbit bone marrow stromal cells (BMSCs) were seeded into 96-well plates (5⊆10^3^ cells/well) for 24 h. The culture medium was then replaced with adCDM-CM (200 µL/well) and incubated for 24 and 48 h. Twenty microliters of CCK-8 reagent (C0038, Beyotime) were added to each well and incubated for 2 h. The optical density (OD) of each well was measured using a microplate reader at 450 nm. Medium without adCDM-CM was used as the negative control, and medium with 0.5 % phenol was used as the positive control. Three independent experiments were performed in triplicate. Cell viability was calculated using the following formula: cell viability (%) = (OD of adCDM-CM group/OD of negative control group) × 100 %. Cell viability was then scored according to the classification of Ahrari et al. [27], with more than 90 % cell viability: no cytotoxicity; 60–90 % cell viability: slight cytotoxicity; 30–59 % cell viability: moderate cytotoxicity; and less than 30 % cell viability: severe cytotoxicity.

### Hemolysis test

Hemolysis tests were carried out according to the procedures reported by Cao et al. [28]. The adCDM extract was obtained by immersing the scaffolds in sterile saline solution (0.1 g/mL in dry weight). The extract was then incubated at 37 °C for 24 h. The hemolysis ratio was calculated using the formula shown below, by which the biosafety of adCDMs for hemolysis was determined according to the ISO standard (eligible if hemolysis ratio was < 5%). Saline-only and double‑distilled water were used as negative and positive controls, respectively.

$$ \mathrm{Hemolyctic}\kern0.5em \mathrm{ratio}=\frac{\mathrm{A}\kern0.5em \mathrm{value}\kern0.5em \mathrm{of}\kern0.5em \mathrm{experimental}\kern0.5em \mathrm{group}\kern0.5em \hbox{-} \kern0.5em \mathrm{A}\kern0.5em \mathrm{value}\kern0.5em \mathrm{of}\kern0.5em \mathrm{NC}\kern0.5em \mathrm{group}}{\mathrm{A}\kern0.5em \mathrm{value}\kern0.5em \mathrm{of}\kern0.5em \mathrm{PC}\kern0.5em \mathrm{group}\kern0.5em \hbox{-} \kern0.5em \mathrm{A}\kern0.5em \mathrm{value}\kern0.5em \mathrm{of}\kern0.5em \mathrm{NC}\kern0.5em \mathrm{group}}\kern0.5em \times 100\% $$

### Pyrogen test

The rabbit pyrogen test was performed in healthy male New Zealand white rabbits according to the protocol described by Yamamoto et al. [29]. The adCDM extract (10 mL/kg) was injected once into the auricular vein in three rabbits. The rectal temperature was measured six times at 30-minute intervals for three hours after injection. The sample was judged to be a negative control when the total increase in body temperature in the three animals was below 1.3 °C. The sample was judged to be positive when the total increase in body temperature in the three animals was over 2.5 °C.

### Subcutaneous implantation for biocompatibility study (in vivo)

Subcutaneous implantation in a Sprague-Dawley (SD) rat model for test scaffolds was performed to confirm whether the biomaterial composition is non-toxic in a biological milieu [30]. SD rats were randomly distributed between native cartilage and adCDMs to compare the effects of healing. Four weeks later, naked-observation and histopathology analyses were performed to evaluate the *in vivo* biocompatibility of scaffolds in terms of implantation effect on vital organ functions and tissue response near the implant site.

### Establishments of cartilage defect models and treatments with scaffolds

Different animal models, including murine, rabbit, ovine, canine, porcine, caprine, equine, can be used in preclinical trial of OCDs [31]. Rabbit models was selected for our study due to relatively low cost, requirement of simple husbandry. In additional, rabbit can reach early skeletal maturity at 9 months and have a long track record of biomedical research. A 3 mm diameter has been considered previously the critical sized defect to prevent spontaneous healing [31]. Thus, a total of 24 adult male New Zealand white rabbits (9 weeks, 2.0–2.5 kg) were used in this study. The animals were housed in metal wire cages in a temperature-controlled room under a 12:12 h light-dark cycle at 22–24 °C and 50–60 % relative humidity. They were fed *ad libitum* with standard laboratory chow and tap water. The 48 bilateral knees of the 24 rabbits were randomly divided into three experimental groups of 16 knees per group. Each animal was sedated by intramuscular injection of ketamine hydrochloride (60 mg/kg) and xylazine (6 mg/kg). Under sterile conditions, medial para-patellar arthrotomy was performed in both knees. A full-thickness cylindrical cartilage defect of 4 mm in diameter and 3 mm in depth was created in the patellar groove using a standard-size stainless biopsy punch [32]. The joints were thoroughly rinsed with sterile saline solution before transplantation.

In group I, the defects were left unfilled (negative control). In group II, the defects were filled with pdCDMs-gels (dose, 40 µL for each joint). In group III, the defects were filled with adCDMs-gel (dose, 40 µL for each joint). The rabbits were housed in separate cages and allowed unrestricted activity after surgery.

### Gross morphology

The treatment period of 2 months was determined using the control scaffold (pdCDMs) in our preliminary experiment (data not shown). In this study, four rabbits were randomly selected and sacrificed with an overdose of anesthesia at 1 and 2 months after treatment with the scaffolds. The distal parts of the femurs were excised, photographed, and graded for cartilage repair according to the International Cartilage Repair Society Score (ICRS) macroscopic assessment scores (Additional file 1.Table S1) [32].

### Histological and immunohistochemical analysis

Histological and immunohistochemical analyses were conducted after gross evaluation [32, 33]. Samples were fixed in 4 % paraformaldehyde for 7 days, decalcified in 10 % EDTA for 3 weeks, embedded in paraffin, and cut perpendicularly into 5 μm sections. The sections were then stained with hematoxylin and eosin (H&E) and Masson stains to estimate the cartilaginous matrix distribution. For immunohistochemical analysis, heat-induced epitope retrieval was performed in citrate buffer (P0081, Beyotime) and immunolabeled with primary antibodies (Bsm-33,409 M, Bioss) at 4 °C overnight, followed by incubation with a secondary antibody (Bs-0377R-HRP, Bioss) to detect immunoactivity. Isotype-matched negative control antibodies were used under identical conditions. Finally, all sections were analyzed under a microscope.

### Data analysis

All experiments in this study were performed at least in triplicate for each control and treatment group. The numeric data are expressed as the mean ± SD. Differences between the groups were evaluated using Student’s t-test. Statistical significance was set at P < 0.05. GraphPad Prism 7 (version 7.00, California, USA) (www.graphpad.com) was used for the data analysis.

## Results and Discussions

### Preparation and characterization of scaffolds

Antler cartilage tissue was selected because of its unique cartilage structure [34], regenerating properties [35], and large volume [36]. Both adCDMs and pdCDMs were prepared according to an optimized method described in other studies [19, 20]. Optimization of conditions for scaffold preparation was conducted in three aspects, including homogenization methods, times, and temperature of freezing-thawing. The results showed that homogenization of minced cartilage samples was better performed by a homogenizer to crush them into 1–2 mm particles. The number of freeze-thaw cycles was modified from 2 to 6 times, and the working temperature was changed from − 20 °C to -80 °C.

**Fig. 1:**
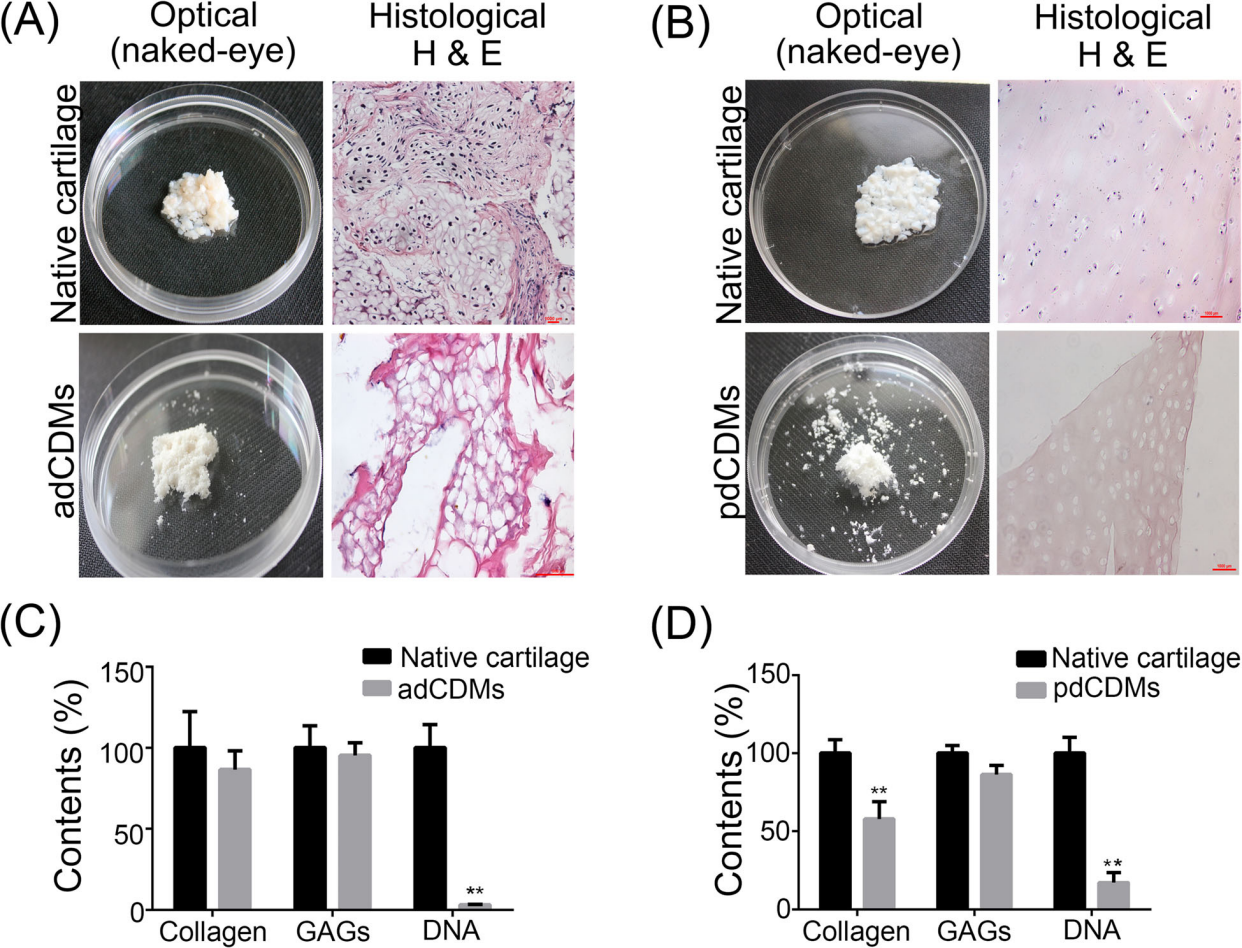
Preparation and characterization of deer antler and porcine joint decellularized cartilage-derived matrix scaffolds (dCDMs). The gross morphology and H&E staining of antler dCDMs (adCDMs) (**A**) and porcine dCDMs (pdCDMs) (**B**). Chemical composition analysis of adCDMs (**C**) and pdCDMs (**D**). **, *p* < 0.05. The untreated cartilage (antler cartilage and porcine joint cartilage) served as the controls. Bar = 1000 μm

Both adCDMs and pdCDMs were loose with a pure white color and became sponge-like materials after freeze-drying treatment (Fig. 1 A & 1B). All area of the adCDMs were deeply stained with cartilage-specific alcian blue, which indicated that no significant non-cartilaginous (such as mesenchyme or clarified cartilage) tissues contamination in sampling (Additional file 2. Figure S1). dCDMs must always be identified by histological staining and quantification of DNA remnants [37]. H&E staining assays showed that cell nuclei-specific dark-purple staining appeared ubiquitously in the cartilage of deer antlers and porcine joints, while few of them were found in adCDMs and pdCDMs (Fig. 1 A & 1B). In additional, DNA concentration of adCDMs and pdCDMs was reduced significantly by 96.82 % ± 0.27 and 82.83 % ± 6.46 %, respectively, (Fig. 1 C & 1D) and the residual DNA was 6.72 ng per mg in the adCDMs group. Insufficient decellularization may cause undesired immune responses, which can influence *in vivo* results [38]. For clinical applications, it is critical to completely remove allogenic/xenogeneic DNA and cell membrane residues. If the residual DNA amount is less than 50 ng per mg of dCDMs, there would be no harmful effects on the immune response [39].

Cartilage is composed of type II collagen and proteoglycans [40]. We confirmed that the adCDMs retained approximately 95.28 % of sulfated GAG and 86.61 % of collagen content compared to native cartilage. In the pdCDMs group, GAG and collagen content accounted for 86.25 % and 57.79 % of porcine joint cartilage, respectively. It should be noted that the collagen content of pdCDMs was significantly decreased compared to that of porcine joint cartilage (*p* < 0.05).

### Evaluation of biosafety and biocompatibility of the scaffolds

To evaluate the scaffolds’ bio-safety and biocompatibility as artificial articular cartilage, a series of *in vitro* and *in vivo* tests, including pyrogen detection, hemolysis analysis, cytotoxicity evaluation, and subcutaneous implantation experiments were performed.

Pyrogens are fever-inducing substances; hence, testing is mandatory and a critical method to ensure the safety of clinical products [41]. Detection of pyrogen showed that the total warming in the adCDM extract and normal saline group (negative control) was 0.82 and 0.72 °C, respectively. The average warming in the adCDM extract and negative control was 0.41 and 0.38 °C, respectively. During the pyrogen analysis, the temperature of each experimental rabbit did not exceed 0.6 °C (Additional file 3.Table S2), which met the standard requirements [42]. The term “hemolysis” refers to the pathological process of breakdown of red blood cells in blood, which serves as an important cause of clinical problems [43]. Hemolysis analysis in our study showed that few ruptures or agglutination of red blood cells were found in both the negative control and adCDM extract groups, while more of them appeared in the H_2_O group (positive group). The hemolysis rate of the adCDM extract was calculated as 4.40 % (Additional file 4: Table S3), which was eligible according to the ISO standard (< 5 %) [28].

**Fig. 2:**
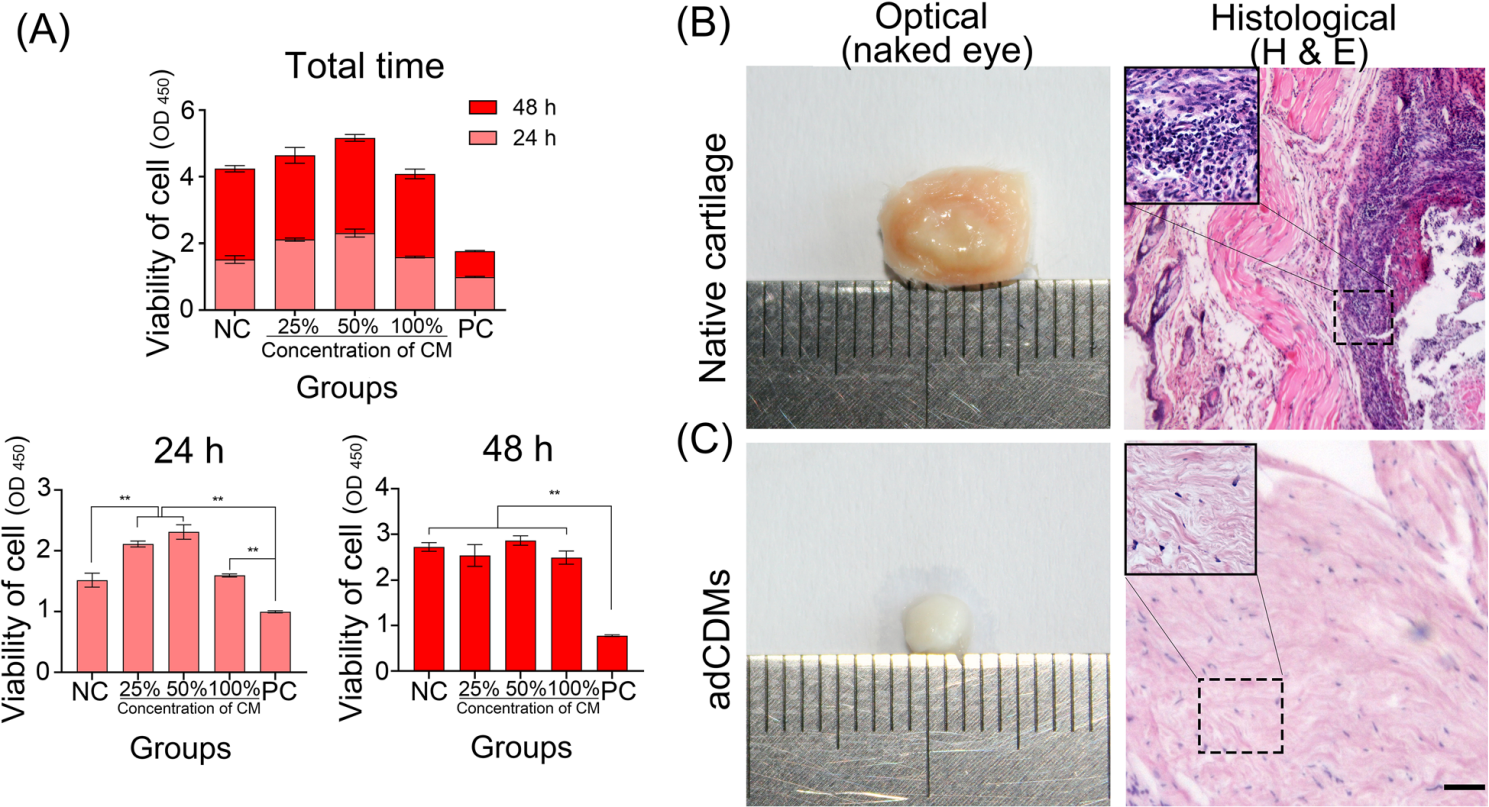
Cytotoxicity analysis and subcutaneous implantation evaluation. (**A**) Cytotoxicity analysis of conditioned medium (CM) of the antler decellularized cartilage-derived matrix scaffold (adCDMs). The gross morphology and H&E staining of both native cartilage (**B**) and adCDMs (**C**) in subcutaneous implantation evaluation assay. Bar = 500 μm

Few cytotoxic chemicals (such as SDS) may still remain in the adCDMs after processing procedure, which would affect the subsequent animal experiments. At time point of 24 h, the abundance of both culture medium with 25 % CM and 50 % CM were significantly higher than that of negative control (NC) and positive control (PC) (p < 0.01) (Fig. 2 A). Group with full concentration of CM (100 % CM) was equally to the NC, while it was significantly higher than that of PC (*p* < 0.01) (Fig. 2 A). At time point of 48 h, there were no significant differences among the group NC, 25 %CM, 50 % CM and 100 %CM. Full area growth of cells in the plate can cause contact inhibition, which could arrest the growth of the cells [44]. In conclusion, cytotoxicity analysis of the CM of the adCDMs showed that over 100 % adCDMs-CM in medium had no toxic effects on the growth of rabbit BMSCs (Fig. 2 A), according to the classification (over 90 % percentage of negative control) of Ahrari et al. [27]. Interestingly, in this study, in addition to the non-cytotoxic properties demonstrated with cultured BMSCs, the adCDMs showed a trend of improving of proliferation of the BMSCs during a 24-h period (Fig. 2 A). The cell proliferation-promoting effects of molecules from adCDMs could contribute to osteochondral repair [11].

A subcutaneous implantation experiment was conducted to evaluate the biocompatibility of the adCDMs. There were no obvious changes in the implantation sites, and no adverse reactions such as exudate, pus, or fistula in the operation areas. In addition, animal activity did not change significantly before and after implantation. It is generally accepted that acute inflammation is a direct response of the tissue to implantation on the 7th day [45]. The inflammatory response was mainly located in the outer surface area of the membranes, tissue fluid, and emigration of immunocytes from blood vessels to implantation sites [46]. The results of the three-week experiment showed that the implants in the adCDMs group were cartilage-like white scaffolds (Fig. 2 C). There was no obvious inflammatory adhesion or connective tissue in the surrounding tissues. In the control group (native antler cartilage), the implants were wrapped in a layer of connective tissue with pale-yellow inflammatory adhesions (Fig. 2 B). Histological examination revealed that the adCDM group had no inflammatory cell infiltration or telangiectasia (Fig. 2 C). In the control group, the implant center was wrapped with a layer of necrotic and broken inflammatory cells (Fig. 2 B). Overall, subcutaneous implantation of adCDMs showed very good biosafety and biocompatibility.

The adCDMs-gel and pdCDMs-gel prepared in this project had a translucent milky white appearance after digestion. It was fluid-like at 4 °C and became a jelly-like gel after a 30 min bath at 37 °C (Additional file 5: Figure S2). We found that dCDMs, which fill in the rabbit articular cartilage defect, can quickly gel and adhere to the injured area. Thus, the dCDMs-gels were suitable for the repair of OCDs in preclinical and clinical settings.

### The effects of scaffolds on cartilage defects by morphological observation

Scaffolds are a major component of tissue engineering strategies. Biologic scaffolds derived from decellularized tissues have been successfully used in tissue engineering [47]. In this study, both adCDMs and pdCDMs were implanted into rabbit articular cartilage defects for 2 months to evaluate their therapeutic effects. Photographs of the full-thickness cylindrical cartilage defects and after transplantation using dCDMs-gels are shown in Additional file 6. Figure S3.

**Fig. 3:**
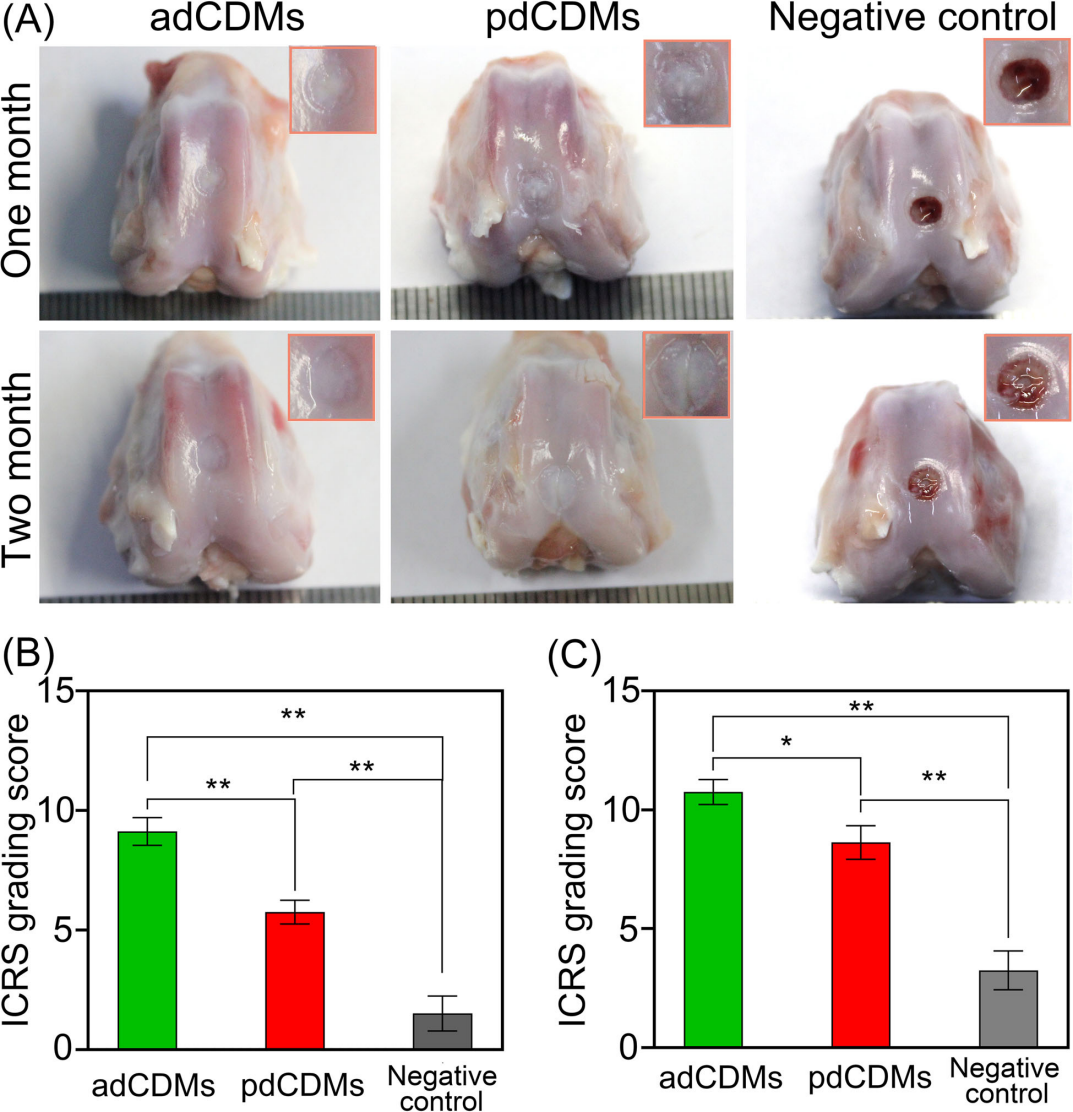
The effects of scaffolds on cartilage defect in rabbits by morphological observation. (**A**) The gross morphology of cartilage defects after treatment by adCDMs for one month and two months. Calculation of ICRS score of cartilage defects for one month (**B**) and two months (**C**). Controls: negative control, and pdCDMs. *, *p* < 0.05; **, *p* < 0.01

Morphological observation in 1 month revealed that the defects in the negative control (normal saline) group did not heal significantly, and a small amount of red fiber-like fillings was found in the central area (Fig. 3 A). In the adCDM and pdCDM groups, the fillings were absorbed and disappeared. The surface of the defect in the adCDM group was smooth and showed a cartilage-like appearance; however, there were visible boundaries between the original tissues and the new tissues (Fig. 3 A). The defect in the pdCDMs group was also filled with new cartilage-like tissue; however, the surface was rough and uneven, and the boundary between the original tissues and the new tissues was obvious (Fig. 3 A). The ICRS score of the adCDMs group (9.13 ± 1.64) was significantly (*p* < 0.01) higher than that of the two controls, the negative control (1.5 ± 2.07) and the pdCDMs group (5.75 ± 1.39) (Fig. 3B). In summary, the ICRS score results were consistent with the results of the naked eye observation.

Two months after the operation, the cartilage defects in the negative group were still filled with red fibrous materials (Fig. 3 A). At the defects in the adCDMs group, the surface of the new cartilage was smooth, the edge transition was gentle, and the appearance (especially the color of newly formed tissue) was the same as that of the original cartilage (Fig. 3 A). The defects of the pdCDM group were covered by white fibrocartilage (white color widely distributed in the middle area of newly formed tissue), and the surface was smooth; however, there was a protruding dent in the central area (Fig. 3 A). We can also found that the difference of repair status between adCDMs and pdCDMs was become smaller in two month than that in the one month. The application of adCDMs still showed better repair effects. In addition, the ICRS score of the adCDMs group (10.75 ± 1.49) was significantly (*p* < 0.01) higher than that of the two controls, negative control (3.25 ± 2.31) and pdCDMs (8.63 ± 2.00) (Fig. 3C), which were consistent with the results of morphological observation by naked eye.

The fast growth of bone tissues in the antler may be due to unique osteogenesis [17]. Antler cartilage contains blood vessels and can regenerate annually [6]. The superior repairing effects of adCDMs on cartilage defects are most likely due to their peculiarities. However, the specific mechanism involved in this process requires further study.

### The histological examination of scaffolds-treated cartilage defects in rabbits for 1 month

An ideal scaffold should help to retain cells in the desired location and provide appropriate biochemical signals in the same way as the natural ECM, which it substitutes [47]. To further evaluate the repair of the defect, we performed a histological examination of the collected rabbit joints.

**Fig. 4:**
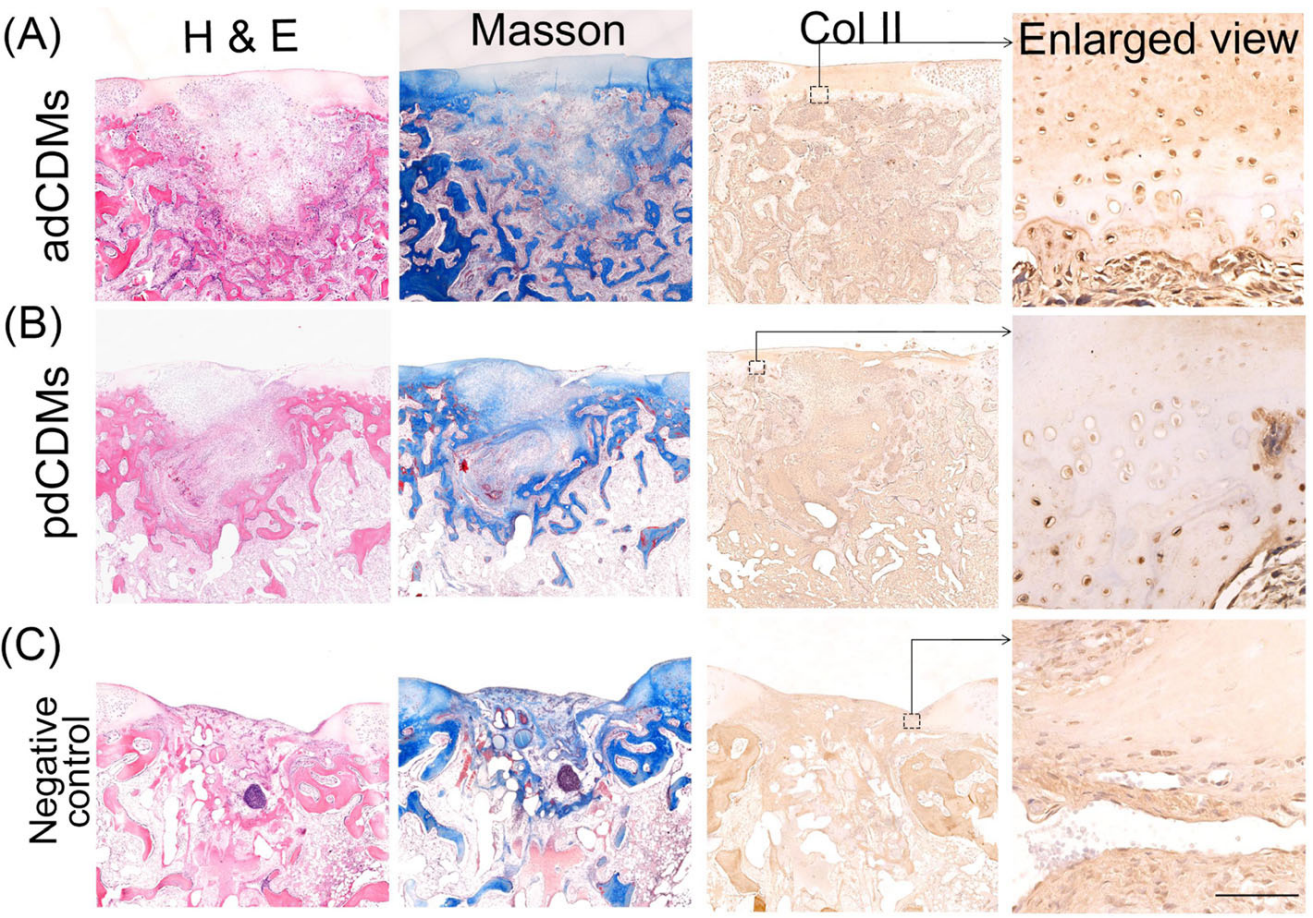
The histological examination of scaffolds-treated cartilage defects for 1 month. (**A**) adCDMs; (**B**) pdCDMs; (**C**) Negative control. H&E: H&E staining; Masson: Masson staining; Col II: Collagen type II immunolocalization. Bar 500 μm

One month after the operation, the surface of the healing cartilage tissue at the adCDM-filled site was flat, and the junction of the newly formed and original cartilage was smooth (Fig. 4 A). Because CDMs consist of native tissue, they can undergo cellular remodeling, which can promote integration with host tissue, and enable them to be degraded and replaced by new tissue over time [48]. In our study, the newly formed chondrocytes were located in the irregular cartilage lacuna, which was mainly located in the lower layer of the cartilage layer (Fig. 4 A). Masson staining results showed deeper blue staining close to the newly formed cartilage, indicating that the new cartilage tissue was hyaline cartilage (Fig. 4 A). In additional, both articular surface and subchondral tissue were deeply stained with the cartilage-specific alcian blue (Additional file 7. Figure S4). Many alcian bule positive staining inside trabecular bone were found (Additional file 7. Figure S4, arrow), which means that the repairing of damaged cartilage by adCDMs was through endochondral osteogenesis. Specific localization of type II collagen in adCDMs was found in cartilage lacunae and the surrounding matrix. Staining was also observed in the subchondral bone tissue (Fig. 4 C). Collagen type II, a cartilage marker, was highly expressed in antler cartilage during antler development [49]. The healed cartilage of the adCDMs group expressed more collagen type II, which may be induced by the special structure of the antler cartilage.

In the pdCDMs group, the repaired cartilage protruded from the joint plane, the junction was broken, and there were cavities inside (Fig. 4B). Masson staining showed uneven blue distribution in new tissues. The articular surface was slightly, while the subchondral tissue were deeply stained with alcian blue (Additional file 7. Figure S4). Same as the adCDMs group (Additional file 7. Figure S4, arrow), the pdCDMs induced repairing of damaged cartilage was also through endochondral osteogenesis. In addition, lighter Col II staining was observed (Fig. 4B).

In the negative control group, defects still existed in the joint plane, and poor transition at the junction with poor-healing cartilage was observed (Fig. 4 C, Additional file 7. Figure S4). Holes, slender fibroblast-like cells, and inflammatory cells were found in the filler, which were randomly arranged. The results of Col II staining were consistent with those found by Masson staining (Fig. 4 C).

### The histological examination of scaffolds-treated cartilage defects in rabbits for 2 months

**Fig. 5:**
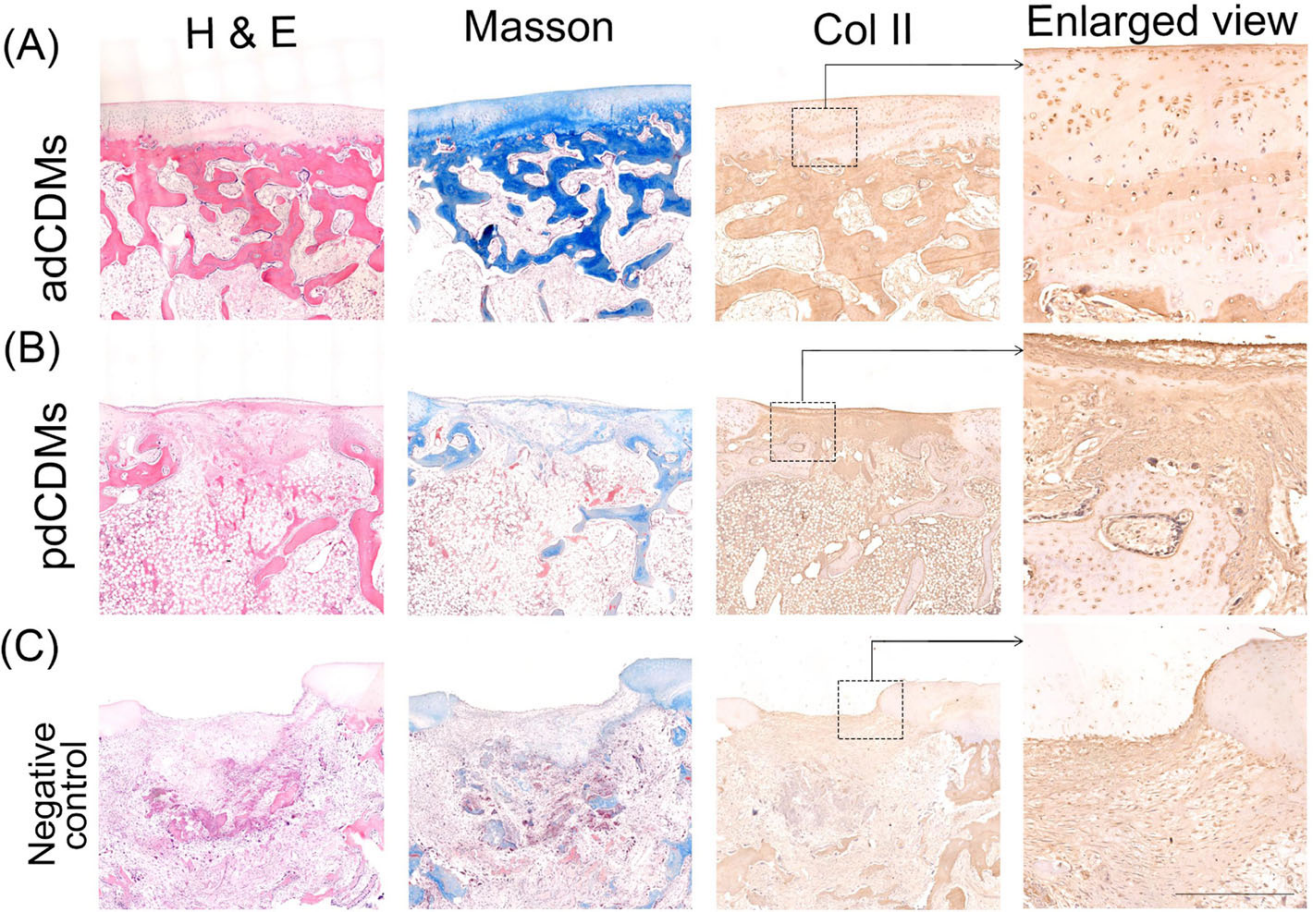
Histologic examination of scaffolds-treated cartilage defects after 2 months. (**A**) adCDMs; (**B**) pdCDMs; (**C**) Negative control. H&E: H&E staining; Masson: Masson staining; Col II: Collagen type II immunolocalization. Bar 500 μm

Two months after the operation, the surface of the healing cartilage was smooth and the repaired cartilage at the adCDM site was completely fused with the original cartilage. No internal joints were found, and the cartilage lacuna in the central area contained an abundance of newly formed cartilage (Fig. 5 A). The ECM was deeply stained with Masson staining (Fig. 5 A). A positive signal for Col II was observed in the cartilage lacuna and ECM (Fig. 5 A). Moreover, the subchondral bone tissue had almost been reconstructed, and the newly formed bone trabecula was interwoven into a mesh (Additional file 8. Figure S5). These results indicate that the repair of cartilage using adCDMs occurs through entochondrostosis.

The surface of the pdCDM group was smooth but with small depressions. The filling cells are mostly long spindle-like fibroblasts with cartilage lacunae interspersed in them (Fig. 5B). A cross-distribution pattern of cartilage lacunae (blue) and red fibrous tissue (pink) was found in newly formed tissues under the articular surface (Additional file 8. Figure S5). Both Masson staining and Col II histochemical results showed that collagen was only distributed in new cartilage in the cartilage matrix around the cells (Fig. 5B). In the negative control group, the defect was still obvious, and the filler was composed of many fibroblast-like cells and had high evidence of inflammation (Fig. 5 C, Additional file 8. Figure S5).

Cartilage is characterised by avascular, aneural structure, cellular arrangement, and dense extracellular structure. The repair and regeneration of cartilage remains unsolved in the clinic [50]. Cells, bio-active molecules, and appropriate scaffolds are crucial to prepare a new cartilage tissue [50]. Among the scaffolds, hyaluronic acid (HA) hydrogel has been extensively investigated for cartilage repair especially in nanomedicine therapy. In additional, various chemical and structural modifications of HA can enhance the functions of HA and its derivatives in tissue engineering [51]. Antlers are structurally comprised internal (cartilage and bone) and external components (skin, blood vessels, and nerves)[52, 53]. The antler cartilage is unique since it is vascularized, and it can regenerate annually. The antler itself has been confirmed to be regenerated through entochondrostosis [52] at an incredible speed (2 cm/d). Our study is the first to report the use of deer antler cartilage as well as adCDMs for the repair of OCDs.

Nowadays, we found that when materials at the nanoscale level, it behave very differently compared to larger scales and nanomaterials show unique physical and chemical properties in comparison to their bulk form [54]. We will performed the proteomics technology to find the special chondrogenesis stimulating molecules between the adCDMs and pdCDMs, and integrate them with nanomaterials (such as HA) for improving the functionality of cartilage repair of OCDs in the future.

## Conclusions

In this study, adCDMs were produced by combining freezing-thawing and enzymatic degradation with optimization. Our developed adCDMs were rich in collagen and GAGs and were devoid of cells. Good biosafety and biocompatibility were also observed, which suggests that the adCDMs were suitable for preclinical and clinical applications. During the four- and eight-week treatments, the cartilage at the adCDMs sites healed well, and the ICRSs were significantly higher than those of controls (pdCDMs and normal saline) (*p* < 0.05). The ECM of the adCDM group was deeply stained using Masson staining. Col II positive signals were present in the cartilage lacuna and ECM. Moreover, the subchondral bone tissue had almost been reconstructed, and the newly formed bone trabecula was interwoven into a mesh. In conclusion, adCDMs could achieve good cartilage regeneration in a rabbit knee OCDs model, providing a new approach for the development of cartilage regeneration materials.

## Supplementary Information

**Additional file 1:Table S1**: ICRS macroscopic evaluation of cartilage repair.

**Additional file 2: Figure S1**: Histological analysis of adCDMs by using alcian blue staining.

**Additional file 3: Table S2**:Temperature monitoring in pyrogen analysis.

**Additional file 4: Table S3**:Hemolysis analysis

**Additional file 5: Figure S2**: Photograph of adCDMs at 4°C (A) and after a 30-minute bath at 37°C (B).

**Additional file 6: Figure S3**: Photograph of the full-thickness cylindrical cartilage defects and after trans-plantation using dCDMs-gels.

**Additional file 7: Figure S4**. The histological examination by using alcian blue staining and H&E staining of scaffolds-treated cartilage defects for 1 month.

**Additional file 8: Figure S5**. The histological examination by using alcian blue staining and H&E staining of scaffolds-treated cartilage defects for 2 month.

## Data Availability

The raw data used and/or analyzed during the current study are available from the corresponding author on reasonable request.

## Abbreviations

OCDs: Osteochondral defects
AnSCs: Antler stem cells
GAGs: Glycosaminoglycans
CDMs: Cartilage-derived matrix scaffolds
ECM: Extracellular matrix
adCDMs: Antler decellularized CDMs
pdCDMs: Porcine decellularized CDMs
HA: Hyaluronic acid
