# Supplementary material for: The use of a novel deer antler decellularized cartilage-derived matrix scaffold for repair of osteochondral defects
Source: J Biol Eng. 2021 Sep 3;15:23. doi: 10.1186/s13036-021-00274-5 (PMC8414868; doi:10.1186/s13036-021-00274-5)
Supplement: Supplementary file 1 — Additional file 1:Table S1: ICRS macroscopic evaluation of cartilage repair. [file 13036_2021_274_MOESM1_ESM.pdf]

Additional file 1: Table S1: ICRS macroscopic evaluation of cartilage repair

**Categories**	**Score**
Degree of defect repair	
In level with surrounding cartilage	4
75% repair of defect depth	3
50% repair of defect depth	2
25% repair of defect depth	1
0% repair of defect depth	0
Integration to border zone	
Complete integration with surrounding cartilage	4
Demarcating border <1 mm	3
3/4 of graft integrated, 1/4 with a notable border >1 mm width	2
1/2 of graft integrated with surrounding cartilage, 1/2 with a notable border >1 mm	1
From no contact to 1/4 of graft integrated with surrounding cartilage	0
Macroscopic appearance	
Intact smooth surface	4
Fibrillated surface	3
Small, scattered fissures or cracks	2
Several, small or few but large fissures	1
Total degeneration of grafted area	0
Overall repair assessment	
Grade I: normal	12
Grade II: nearly normal	11-8
Grade III: abnormal	7-4
Grade IV: severely abnormal	3-1
