# Supplementary material for: The use of a novel deer antler decellularized cartilage-derived matrix scaffold for repair of osteochondral defects
Source: J Biol Eng. 2021 Sep 3;15:23. doi: 10.1186/s13036-021-00274-5 (PMC8414868; doi:10.1186/s13036-021-00274-5)
Supplement: Supplementary file 2 — Additional file 2: Figure S1: Histological analysis of adCDMs by using alcian blue staining. [file 13036_2021_274_MOESM2_ESM.pdf]

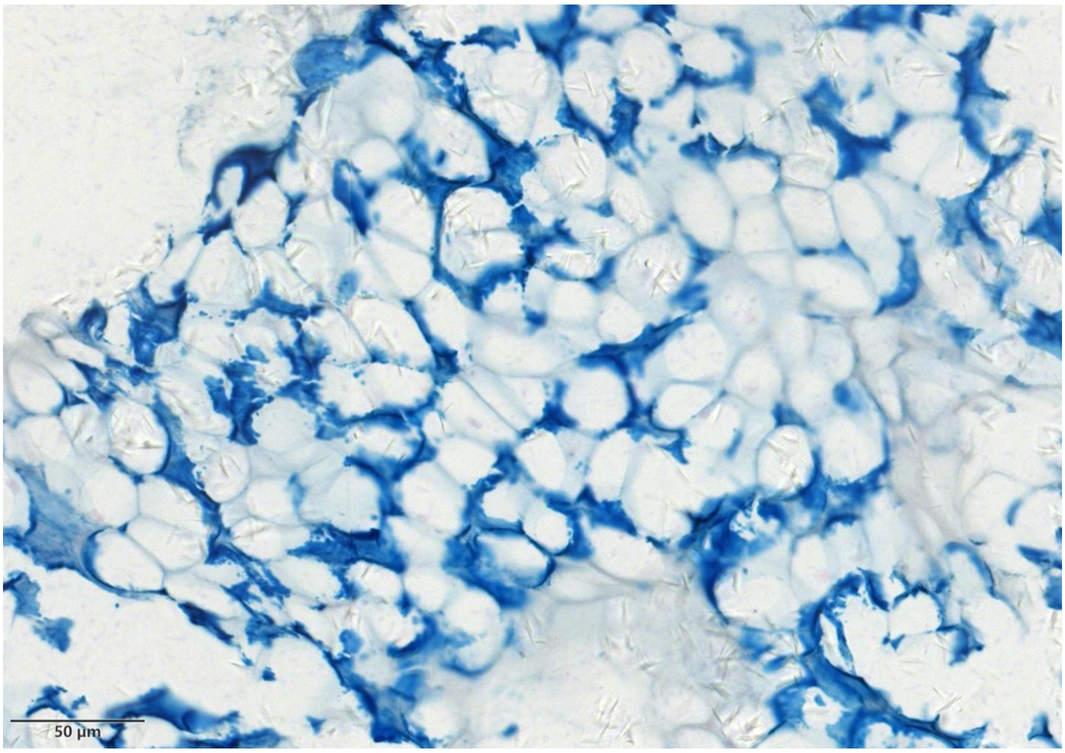

Additional file 2: Figure S1: Histological analysis of adCDMs by using alcian blue staining. Bar = 50 μm.
