# Supplementary material for: The use of a novel deer antler decellularized cartilage-derived matrix scaffold for repair of osteochondral defects
Source: J Biol Eng. 2021 Sep 3;15:23. doi: 10.1186/s13036-021-00274-5 (PMC8414868; doi:10.1186/s13036-021-00274-5)
Supplement: Supplementary file 3 — Additional file 3: Table S2:Temperature monitoring in pyrogen analysis. [file 13036_2021_274_MOESM3_ESM.pdf]

Additional file 3: Table S2:Temperature monitoring in pyrogen analysis

Group	Body weight(g)	Initial temperature (°C)	Warming (°C)	Total warming (°C)
adCDMs extract	564.40 ± 14.40	38.73 ± 0.52	0.41 ± 0.11	0.82 ± 0.20
Negative control (normal saline)	567.40 ± 7.50	38.91 ± 0.55	0.38 ± 0.10	0.72 ± 0.33
