# Supplementary material for: The use of a novel deer antler decellularized cartilage-derived matrix scaffold for repair of osteochondral defects
Source: J Biol Eng. 2021 Sep 3;15:23. doi: 10.1186/s13036-021-00274-5 (PMC8414868; doi:10.1186/s13036-021-00274-5)
Supplement: Supplementary file 4 — Additional file 4: Table S3:Hemolysis analysis [file 13036_2021_274_MOESM4_ESM.pdf]

Additional file 4: Table S3:Hemolysis analysis

Groups	Absorbance, OD_{545}					Average（OD 545）	Hemolysis ratio（%）
Positive control（ddH_2O）	0.127	0.126	0.13	0.127	0.128	0.127	
Negative control（normal saline）	0.04	0.041	0.041	0.042	0.041	0.041	
adCDMs extract	0.044	0.044	0.045	0.045	0.045	0.044	4.40%
