# Supplementary figures and images for: The use of a novel deer antler decellularized cartilage-derived matrix scaffold for repair of osteochondral defects
Source: J Biol Eng. 2021 Sep 3;15:23. doi: 10.1186/s13036-021-00274-5 (PMC8414868; doi:10.1186/s13036-021-00274-5)

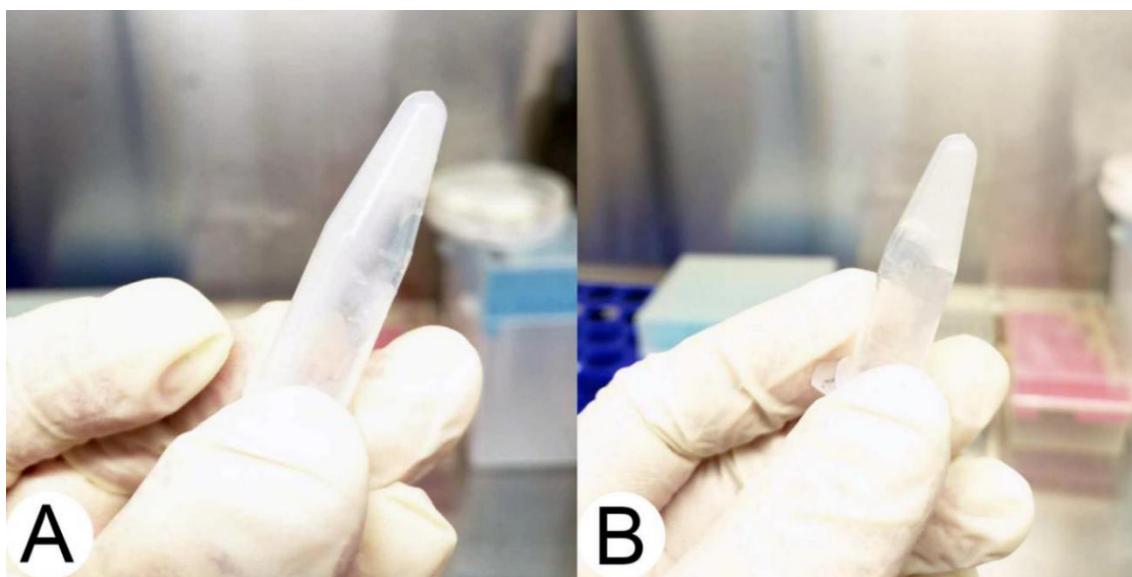

**Additional file 5: Figure S2:** Photograph of adCDMs at 4°C (A) and after a 30-minute bath at 37°C (B).

Supplement: Supplementary file 5 — Additional file 5: Figure S2: Photograph of adCDMs at 4°C (A) and after a 30-minute bath at 37°C (B). [file 13036_2021_274_MOESM5_ESM.pdf]
