# Supplementary material for: The use of a novel deer antler decellularized cartilage-derived matrix scaffold for repair of osteochondral defects
Source: J Biol Eng. 2021 Sep 3;15:23. doi: 10.1186/s13036-021-00274-5 (PMC8414868; doi:10.1186/s13036-021-00274-5)
Supplement: Supplementary file 6 — Additional file 6: Figure S3: Photograph of the full-thickness cylindrical cartilage defects and after trans-plantation using dCDMs-gels. [file 13036_2021_274_MOESM6_ESM.pdf]

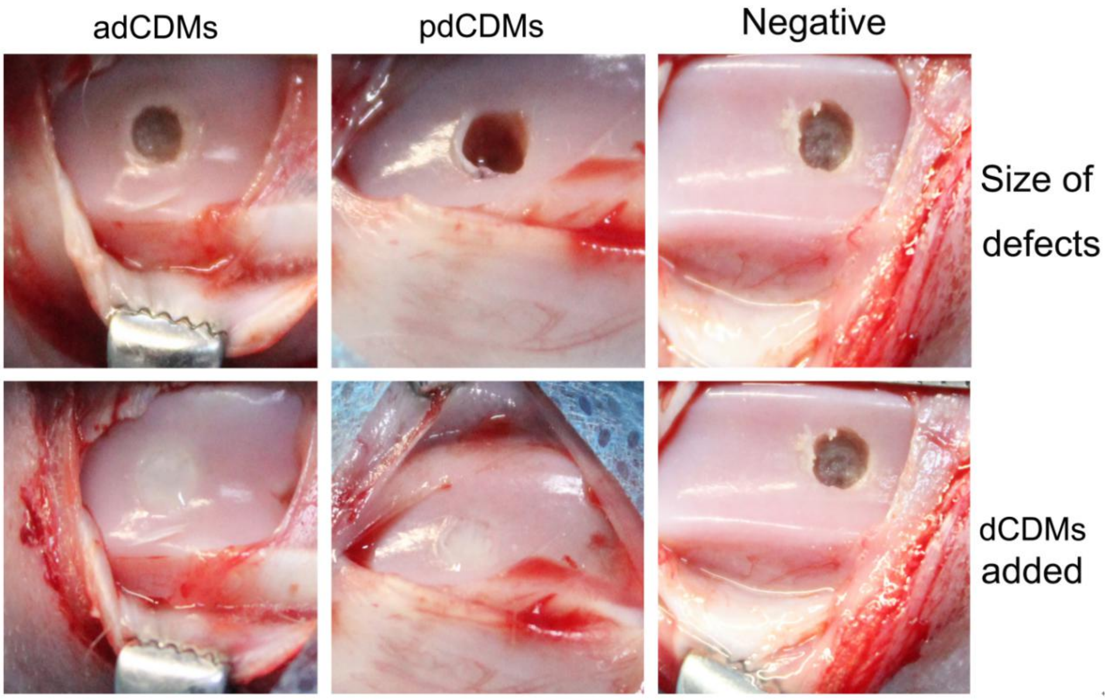

Additional file 6: Figure S3: Photograph of the full-thickness cylindrical cartilage defects and after transplantation using dCDMs-gels.
