# Supplementary material for: The use of a novel deer antler decellularized cartilage-derived matrix scaffold for repair of osteochondral defects
Source: J Biol Eng. 2021 Sep 3;15:23. doi: 10.1186/s13036-021-00274-5 (PMC8414868; doi:10.1186/s13036-021-00274-5)
Supplement: Supplementary file 7 — Additional file 7: Figure S4. The histological examination by using alcian blue staining and H&E staining of scaffolds-treated cartilage defects for 1 month. [file 13036_2021_274_MOESM7_ESM.pdf]

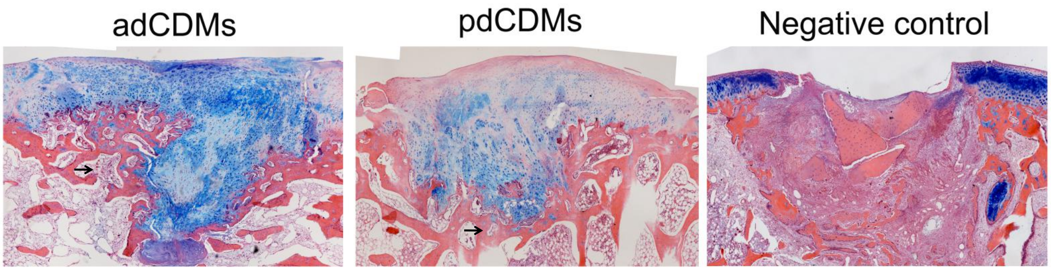

Additional file 7: Figure S4. The histological examination by using alcian blue staining and H&E staining of scaffolds-treated cartilage defects for 1 month.
